# Extracts from *Pulsatilla patens* target cancer-related signaling pathways in HeLa cells

**DOI:** 10.1038/s41598-021-90136-3

**Published:** 2021-05-20

**Authors:** Grażyna Łaska, Magdalena Maciejewska-Turska, Elwira Sieniawska, Łukasz Świątek, David S. Pasco, Premalatha Balachandran

**Affiliations:** 1 Department of Agri-Food Engineering and Environmental Management, Bialystok University of Technology, 15-351 Bialystok, Poland; 2 Department of Pharmacognosy, Medical University of Lublin, 20-093 Lublin, Poland; 3 Department of Virology With SARS Laboratory, Medical University of Lublin, 20-093 Lublin, Poland; 4 National Center for Natural Products Research, School of Pharmacy, University of Mississippi, University, Oxford, MS 38677 USA

**Keywords:** Analytical chemistry, Medicinal chemistry, Cancer, Molecular biology

## Abstract

The purpose of this study was to determine if a methanolic extract of the *Pulsatilla patens* (L.) Mill. can inhibit the progression of cancer through the modulation of cancer-related metabolic signaling pathways. We analyzed a panel of 13 inducible luciferase reporter gene vectors which expression is driven by enhancer elements that bind to specific transcription factors for the evaluation of the activity of cancer signaling pathways. The root extract of *P. patens* exhibited strong inhibition of several signaling pathways in HeLa cells, a cervical cancer cell line, and was found to be the most potent in inhibiting the activation of Stat3, Smad, AP-1, NF-κB, MYC, Ets, Wnt and Hdghog, at a concentration of 40 µg/mL. The methanolic extracts of *P. patens* enhanced apoptotic death, deregulated cellular proliferation, differentiation, and progression towards the neoplastic phenotype by altering key signaling molecules required for cell cycle progression. This is the first study to report the influence of *Pulsatilla* species on cancer signaling pathways. Further, our detailed phytochemical analysis of the methanolic extracts of the *P. patens* allowed to deduce that compounds, which strongly suppressed the growth and proliferation of HeLa cancer cells were mainly triterpenoid saponins accompanied by phenolic acids.

## Introduction

Cervical cancer is the third most common cancer as well as one of the leading causes of cancer-related death of women worldwide^1^. It causes about 13% of cancer cases in women. Cervical cancer epidemiology studies show that it is a serious oncological problem in developing countries, where 85% of the 500,000 cases are diagnosed diseases in the Word^2^. According to statistics in the world, the highest risk of disease is observed in Africa and South America, and in Europe, it is Romania, Bulgaria, and Serbia. The lowest incidence and mortality from cervical cancer occur in Australia, New Zealand, the United States, and Canada. About 60% of cases of this cancer occur in women aged 45–64^2^.

The first human cell line was obtained by George Gey in 1951 from a biopsy of cervical cancer, and was later named HeLa after the patient, Henrietta Lacks. Currently, these cells are one of the most frequently used cell lines in scientific research^3^. HeLa cells are aneuploid and divide rapidly^4^. These cells have a special character, resulting from altered chromosome number and structural disorder, compared to normal cells, which especially applies to chromosome 11, 5, 19, X^3,5^. Studies of the HeLa genome confirmed the expression of genes responsible for the metabolic pathways connected with DNA repair and the cell cycle. Research by other authors has shown that chromothripsis occurs in 2–3% of all cancers^6^. It is believed, that chromothripsis and loss of heterozygosity, LOH on chromosome 11 contributed to the development of HeLa cervical cancer^7^. The use of the HeLa cell line allowed for the development of inactivated polio vaccine (IPV) by J. Salk in 1952^8^, description of human immunodeficiency virus (HIV) infection mechanism by R. Axel and E. Robey in 1986^9^, and the role of the human papillomavirus (HPV) in cancer transformation by H. Hansen in 1984^10^. Cervical dysplasia and cancer transformation may be the result of infection with the human papillomavirus (HPV). Hausen in 1982 proved that chronic HPV infection plays the role of a promoter of carcinogenesis of this tumor^11^, but other risk factors include early sexual intercourse, a large number of sexual partners, numerous pregnancies and deliveries at an early age, hormonal contraception, smoking tobacco and low economic status^11^. Currently, more than 200 types of HPV viruses have been detected. Of these, at least 85 types have been well known^12^. Due to their oncogenic potential, HPV viruses were divided into 3 groups: high, low and possibly low risk. Among the high-risk HPV types, types 16 and 18 account for approximately 80% of all cervical cancer cases^12^. The oncogenic potential of some types of HPV results from the action of the E6 and E7 oncoproteins with transforming properties in the host cells. They disrupt tumor suppressor pathways and are essential for the proliferation of cervical cancer cells. Both oncoproteins are present in 99% of these cells^13^.

Natural products from *Pulsatilla* species have been used for centuries in traditional Chinese and Korean medicine for the treatment of many diseases and ailments. They are known as herbal drugs used for homeopathic treatment of neuralgia, insomnia, bronchitis, coughs, asthma, stress, anxiety, tension, skin eruptions, rheumatism, headaches, earache, eye ailments, hyperactivity, bacterial skin infections and malaria^14^. The *Pulsatilla* genus is composed of 38 species distributed in the northern hemisphere. *Pulsatilla patens* (L.) Mill. belongs to the Buttercup family (Ranunculaceae) commonly known as American Pasqueflower (synonym *Anemone patens* L.) but its chemical composition has not been extensively studied. *P. patens* is a lowland species of Boreo-meridional-continental distribution and it is native to Europe, Russia, Mongolia, China, Canada and United States^15^.

A complex network of signaling pathways is responsible for cellular proliferation, behavior and death in multicellular organisms. Research for the discovery of drugs targeting cancer has gained tremendous importance in the last few decades. Recently, transcription factors (Stat3, Smad, AP-1, NF-κB, E2F, MYC, Ets, Notch, FoxO, Wnt, Hdghog, miR-21, k-Ras) have attracted a fair share of attention as a novel drug target for cancer treatment. These are key transcription factors that regulate cytological processes, including differentiation, cell death, proliferation, oncogenic transformation and apoptosis^16^. Of the several hundred cancer-associated genes and driver gene mutations identified thus far, the vast majority belong to approximately 13 + different signal-transduction pathways. In our research, we have collected a panel of 13 inducible luciferase reporter gene vectors (Stat3, Smad, AP-1, NF-κB, E2F, MYC, Ets, Notch, FoxO, Wnt, Hdghog, miR-21, k-Ras), where expression is driven by enhancer elements that bind to specific transcription factors^17^. These vectors can be used to assess the activity of cancer-related signaling pathways. These are signaling nodes for multiple oncogenic pathways, which transduces intracellular and extracellular signals to the nucleus and control the expression of genes responsible for multiple physiological processes such as cell growth, proliferation, differentiation, positioning, migration, metabolism and apoptosis^16^.

Our study aimed to assess the effect of methanolic extracts from roots and leaves of the *Pulsatilla patens* (L.) Mill. in inhibiting the progression of human cancer cells by selected cancer-related signaling pathways. Different vectors as transcription factors in the signal transduction pathways were studied (Stat3, Smad, AP-1, NF-κB, E2F, MYC, Ets, Notch, FoxO, Wnt, Hdghog, miR-21, k-Ras, pTK – control), in the presence of various inducers (IL-6, TGF-β, PMA, wnt-3a), when cells were exposed to extracts for 4–6 h. Besides, we also performed a detailed phytochemical analysis of this species which allowed for identification of triterpenoid saponins and phenolic acids responsible for the activity.

## Results

### Results of cytotoxicity studies

The methanolic extracts from roots of *P. patens* showed cytotoxicity to all the cell lines included in the assay (Table 1). The IC_50_ concentrations for cytotoxicity for *P. patens* were in the range of 32–58 μg/mL for each cell line indicating a general cytotoxic activity throughout the panel of cancer and non-cancer cells.

**Table 1:** Cytotoxicity activities of *P. patens* methanolic extracts from roots towards a panel of mammalian cell lines.

Sample name	KB^b^	BT-549^c^	SK-OV-3^d^	SK-MEL^e^	HeLa^f^	LLC-PK-1^ g^	Vero^h^
*Cytotoxic activity (IC*_*50*_* µg/mL)*^*a*^							
Root extract P. *patens*	38	32	32	34	58	36	32
Doxorubicin*	1.7	2.2	2.3	1.7	3.9	1.6	> 5

^a^IC_50_ µg/mL is the concentration that affords 50% inhibition of cell growth. ^b-h^ Human cell lines of epidermal carcinoma, breast carcinoma, ovarian carcinoma, skin melanoma, cervical carcinoma, kidney epithelial cells, and kidney fibroblast respectively, *positive control drug.

### The cancer-related signaling pathways in HeLa cells

**Figure 1:**
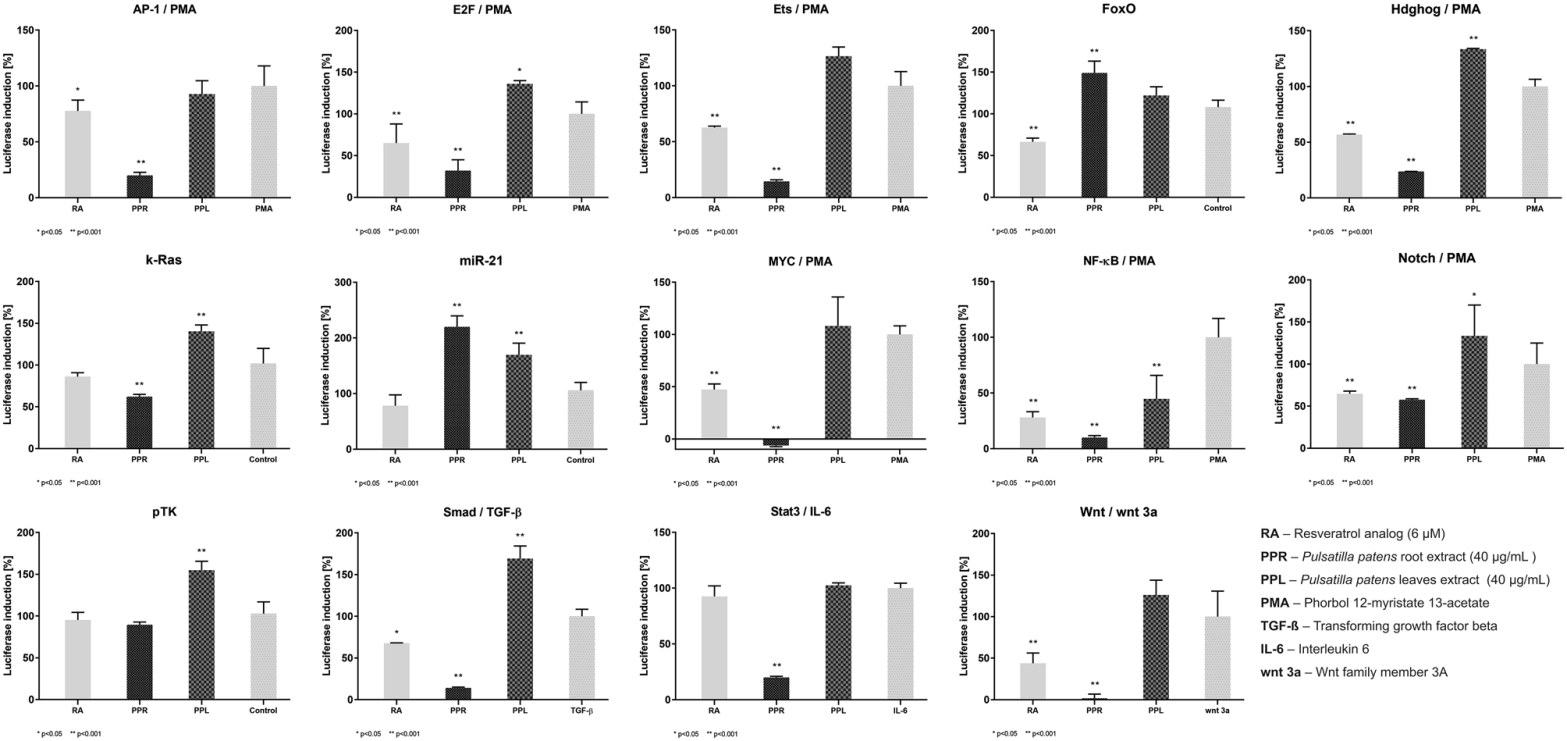
The inhibition of activations of Stat3, Smad, MYC, Ets, E2F, Ap-1, NF-κB, Wnt, Hdghog and pTK-control by methanolic extract of *Pulsatilla patens* and reference/control compound—resveratrol analog. IL-6, TGF-β, PMA, wnt 3a** –** Inducer (promoter) of cancer processes. Resveratrol analog – the analog 3,5,4′-trihydroxy-*trans*-stilbene, *trans*-resveratrol or (*E*)-resveratrol—is a stilbenoid, a type of natural phenol, and a phytoalexin with anticancer activity. The obtained data were statistically analyzed using GraphPad Prism (two-way ANOVA, Dunnett's multiple comparisons test).

The activity of cancer-related signaling pathways was assessed using a panel of luciferase reporter gene vectors, in which luciferase expression is driven by the binding of transcription factors to multiple copies of synthetic enhancers within each vector. The methanolic extracts from the roots of *P. patens* were more powerful than the compounds from leaf extract of this species inhibiting the activation of 11 pathways analysed, with the exception only two, FoxO and miR-21. The active compounds of methanolic extracts from the roots of *P. patens* were found to be the most potent in inhibiting the activation of Stat3, Smad, AP-1, NF-κB, MYC, Ets, Wnt and Hdghog signaling (Fig. 1). E2F and Notch signaling were inhibited stronger by compounds of root extract than leaf extract, but similarly inhibited as by an active antitumor compound used in the research, resveratrol analog. Similarly, the activation of the apoptotic mediators pathways FoxO, miR-21 and k-Ras were not observed with either compounds from the methanolic extracts of *P. patens* (Supplementary Table S1).

The methanolic extracts from the roots of *P. patens* were stronger in potency than active antitumor compound resveratrol analog in each of their concentrations for inhibition of signaling mediated by Stat3, Smad, AP-1, NF-κB, E2F, MYC, Ets, Wnt and Hdghog, but were similar in potency as resveratrol analog for the inhibition of signaling mediated by Notch and k-Ras. However, the resveratrol analog was more effective than active compounds of *P. patens* for FoxO and miR-21. It was found that methanolic extracts from the roots of *P. patens* did not inhibit luciferase expression driven by the minimal thymidine kinase promoter (pTK—control), indicating it as not significant for general cytotoxicity or luciferase enzyme inhibition.

**Figure 2:**
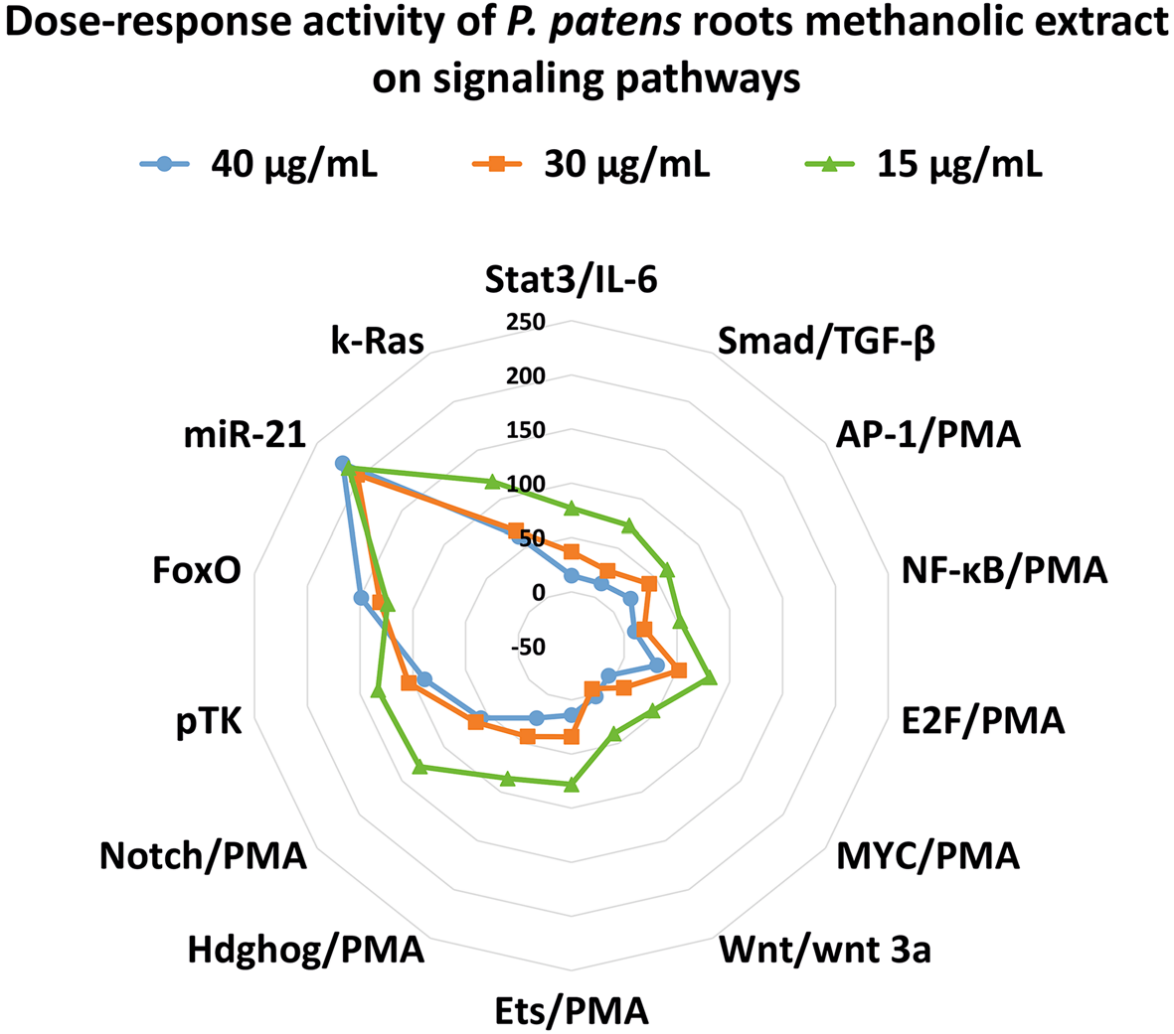
The active compounds from the roots of *P. patens* against cancer-related signaling pathways in HeLa cells with different concentrations of 40 µg/mL, 30 µg/mL and 15 µg/mL.

Since active compounds of methanolic extract from the root of *P. patens* were more forceful than from leaves of this species with respect to inhibiting the activation of pathways, it was determined which concentration of this extract was also a more effective modulator of cancer-related signaling pathways. Active compounds of methanolic extracts from roots of *P. patens* with a concentration of 40 µg/mL were stronger than these compounds from other concentrations (30 µg/mL and 15 µg/mL) (Supplementary Table S1). Active compounds from roots of *P. patens* with a concentration of 40 µg/mL were more potent inhibiting the activation of 12 signaling pathways used, with the exception of only two, FoxO and miR-21. The active compounds from the roots of *P. patens* with a concentration of 40 µg/mL were found to be the most effective in inhibiting the activation of 11 signaling pathways used, such as Stat3, Smad, AP-1, NF-κB, MYC, Ets, Wnt, Hdghog, E2F, Notch and k-Ras (Fig. 2). None of the extracts with the concentrations of 40 µg/mL, 30 µg/mL and 15 µg/mL inhibited the apoptotic mediators FoxO, miR-21 and inhibited the minimal thymidine kinase promoter (pTK control) in the luciferase expression, indicating a weak general cytotoxicity.

**Figure 3:**
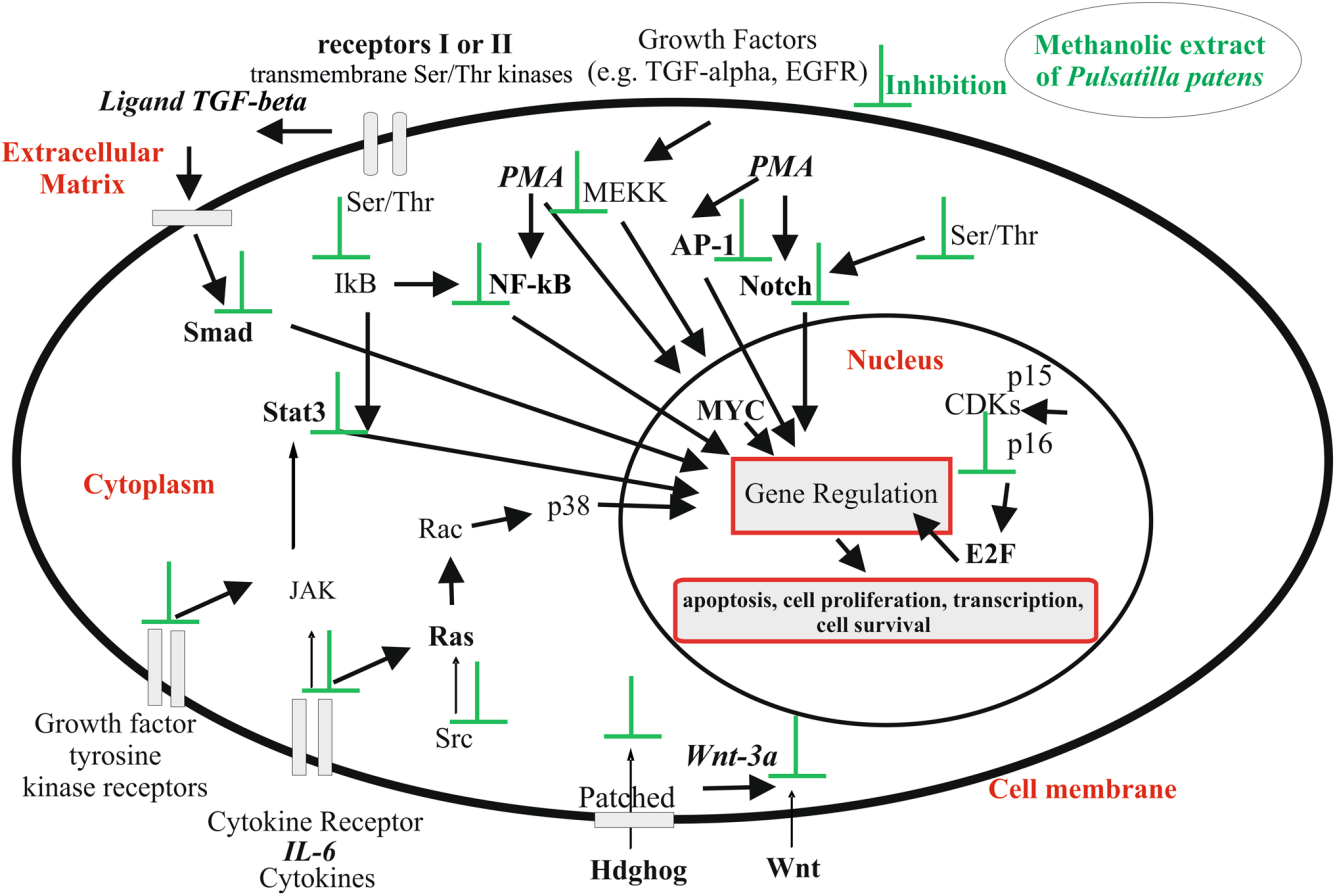
Cancer-related signaling pathways in HeLa cells inhibited by *P. patens* methanolic extract and the mechanism of extract *P. patens* action in the cancer cell. The methanolic extracts of *P. patens* enhance apoptotic death, deregulated cellular proliferation, differentiation, and progression towards the neoplastic phenotype by altering key signaling molecules required for cell cycle progression. *Stat3, Smad, AP-1, NF-κB, E2F, MYC, Notch, Wnt, Hdghog, Ras* Transcription factors, *Cytokines IL-6, Ligand TGF-beta* Inducers, *PMA* Protein kinase activator, phorbol 12-myristate-13-acetate, Wnt-3a protein, *Ser/Thr* Serine/threonine kinases, *EGFR* Epidermal growth factor receptor, *MEKK* Mitogen-activated protein (MAP) kinase, *IkB* Inhibitor of kB, *JAK* Janus kinase, *Src* Protooncogene tyrosine-protein kinase, *Rac* Subfamily of the Rho family of GTPases, *Ras* Ras family kinase, *p38* Mitogen-activated protein kinases, *p15, p16* The cyclin-dependent kinase inhibitors, *CDKs* Cyclins, cyclin-dependent kinases, *Patched* Protein patched homolog.

This is the first report of active compounds of methanolic extracts from roots of *P. patens* from a natural source influencing the cancer-related signaling pathways in HeLa cells, and also the first report of different 13 signaling pathways as transcription factors in cancer processes from the genus *Pulsatilla patens*. Obtained data suggests that the action of *P. patens* methanolic extract is based on the induction of apoptosis and on the inhibition of mitosis by disturbing the HeLa cell cycle (Fig. 3). The small-molecule kinase inhibitors, which, through selectivity and specificity, inhibit the activation of specific proteins, also act on the receptors responsible for oncogenesis. Based on the studied cancer-related signaling pathways in HeLa cells we propose that the methanolic extracts of *P. patens* enhance apoptotic death, deregulated cellular proliferation, differentiation, and progression towards the neoplastic phenotype by altering key signaling molecules required for cell cycle progression. Thereby, this mechanism prevents excessive, harmful proliferation of the HeLa cells and inhibits them.

### The active compounds of methanolic extracts of *P. patens*

The results showed that *P. patens* is a rich source of polyphenolic constituents, mainly saponins and hydroxycinnamic acids. Interestingly, *P. patens* showed a different profile of triterpenoid saponins in comparison to other species from the Ranunculaceae family.

The calibration curves of reference compounds were linear in the range of 0.02–1 mg/mL and 0.002–0.5 mg/mL, for Pulsatilla saponin D and ferulic acid, respectively. The linearity was very good with the correlation coefficient (R^2^) values higher than 0.999, as shown in Table 2. The results obtained in the intraday and interday precision measurements of both reference standards were below 1%, indicating the high stability of the method. The recovery was in the range from 87.5% (RSD 0.89%) for Pulsatilla saponin D to 97.04% (RSD 0.49%) for ferulic acid.

**Table 2:** Method validation parameters for reference substances; linearity, precision, LOD, LOQ and quantification.

Reference substance	Regression equation	*R*^2^	Linearity range (mg/mL)	LOD (mg/mL)	LOQ (mg/mL)	Concentration range (mg/mL)	Intraday precision (*n* = 3) (%)	Interday precision (*n* = 3) (%)
Pulsatilla saponin D	y = 857.12x-99.356	0.9995	0.02–1	0.0461	0.1396	0.05	0.45	0.57
						0.25	0.26	0.11
Ferulic acid	y = 8715.9x + 241.52	0.9998	0.002–5	0.0107	0.0326	0.005	0.76	0.55
						0.01	1.07	0.57

The analyzed compounds vary remarkably with the part of the plant. The total content of phenolic acids was higher in leaves while roots were found to be an abundant source of triterpenoid saponins, representing the dominant class of constituents. The content of saponins was in the range from 0.11 to 2.14 mg/g dw in roots and from 0.14 to 0.32 mg/g dw in the leaves (Table 3; Supplementary Fig. S1). Triterpenoid saponins and phenolic acids present in *P. patens* extracts were characterized and tentatively identified for the first time. Their MS data were listed in Table 4. Most of the triterpenoid saponins found in the studied *P. patens* extracts shared common features. The characteristic solvent adduct ions [M + HCOO]^−^ or deprotonated ions [M‐H]^−^ were usually observed. The composition of sugar residues was deducted based on the fragmentation pathway observed in MS/MS spectra. The peaks with high abundance intensities were formed from the typical for negative ionization mode, simultaneous loss of 470 Da, which was attributed to the cleavage of the ester bond and the elimination of the entire sugar chain, assigned as Glc-Glc-Rha, from C-28 position^18^. Whereas the successive losses of sugar moieties, such as 162 Da (-Glc), 146 Da (-Rha) or 132 Da (-Ara) correspond to the sequence and composition of sugar fragments linked to C-3^16^. According to the literature data, the majority of saponins with a molecular weight higher than 1000 Da found in *Pulsatilla* taxa were determined to be bidesmosidic saponins substituted at both C-3 and C-28 positions with oligosaccharides^19^.

**Table 3:** Content of saponins and hydroxycynnamic acid type compounds in *P. patens* extracts.

COMPOUND	Content (mg/g DW)	
	Leaves	Roots
**Saponins**		
3-*O*-GlcGlcAra, 28-*O*-RhaGlcGlc bayogenin (**22**)	**–**	0.14
3-*O*-GlcGlcAra, 28-*O*-RhaGlcGlc hederagenin (**24**)	**–**	1.10
3-*O*-GlcAra, 28-*O*-RhaGlcGlc bayogenin (**25**)	**–**	0.59
3-*O*-GlcAra, 28-*O*-RhaGlcGlc 23-hydroxybetulinic acid (**27**)	**–**	2.14
3-*O*-Ara, 28-*O*-RhaGlcGlc bayogenin (**28**)	0.23	0.47
3-*O*-GlcAra, 28-*O*-RhaGlcGlc hederagenin (**29**)	0.32	1.84
3-*O*-Ara, 28-*O*-RhaGlcGlc 23-hydroxybetulinic acid (**30**)	0.15	0.97
3-*O*-Ara-Rha, 28-*O*-RhaGlcGlc hederagenin**/**3-*O*-Ara-Rha, 28-*O*-RhaGlcGlc 23-hydroxybetulinic acid (**32**)	0.17	**–**
3-*O*-AraGlc 23-hydroxybetulinic acid (**33**)	**–**	0.12
28-*O*-RhaGlcGlc 23-hydroxybetulinic acid (**36**)	0.16	0.12
3-*O*-AraGlc 23-hydroxybetulinic acid (**37**)	**–**	0.11
3-*O*-Ara, 28-*O*-Glc hederagenin (**41**)	**–**	0.12
3-*O*-Ara, 28-*O*-Glc hederagenin (**42**)	0.14	0.14
**Hydroxycynnamic acid type compounds**		
Dicaffeic acid (**5**)	0.03	0.01
Coumaric acid (**6**)	**–**	0.01
Caftaric acid (**7**)	0.08	**–**
Caffeic acid (**8**)	0.02	0.02
Ferulic acid (**20**)	0.32	0.07
Chicoric acid isomer 1 (**23**)	0.65	0.30
Chicoric acid isomer 2 (**26**)	0.17	0.10
Coumaric acid derivative (**31**)	0.04	**–**

Ara – arabinose; Glc – glucose; Rha – rhamnose; DW – dry weight.

**Table 4:** Chemical composition of *P. patens* extracts.

Comp. No	Tentative identification	Rt (min)	Molecular fromula	MW	[M—H]^-^	Fragments (m/z)	Roots	Leaves	References
1.	Caffeic acid hexoside	1.622	C15H18O9	342.0947	341.0947	179.0559; 161.0539; 135.0455	+	+	^29,60^
2.	Dihydroxybenzoic acid hexoside	6.303	C13H16O9	316.0766	315.0766	153.0567; 109.0337	+	+	^27,31^
3.	Hydroxymelilotic acid	8.988	C9H10O4	182.0544	181.0544	163.0402; 135.0487	+	+	−
4.	Hydroxybenzoic acid isomer 1	9.656	C7H6O3	138.0276	137.0276	119.0236; 108.0210; 93.0393	+	+	^27^
5.	Dicaffeic acid	10.471	C15H18O9	342.0947	341.0947	179.0361; 161.0223; 135.0455	+	+	^60^
6.	Coumaric acid	13.002	C9H8O3	164.0433	163.0428	149.0126; 135.0450; 119.0503	+	−	^60^
7.	Caftaric acid	13.07	C13H12O9	312.0457	311.0457	179.0332; 149.0079; 135.0406; 112.9989	−	+	^30^
8.	Caffeic acid	16.745	C9H8O4	180.0392	179.0392	135.0456; 107.05	+	+	^27,28^
9.	Anemonin	17.621	C10H8O4	192.0391	191.0391	147.0470; 101.0357	+	−	^61^
10.	Vanilic acid	18.505	C8H8O4	168.0385	167.0385	152.0090; 108.0228	+	+	^30^
11.	Ferulic acid deriv	19.544		226.0810	225.0810	193.0516; 135.0475	−	+	^27^
12.	Unknown	19.549		682.2496	681.2496	519.1772; 357.1296; 151.0426	+	−	−
13.	Ferulic acid glucoside isomer 1	20.886	C16H20O9	356.1097	355.1097	193.0508	+	+	^27^
14.	Ferulic acid glucoside isomer 2	21.840	C16H20O9	356.1097	355.1097	193.0508	−	+	^27^
15.	Caffeic acid syryngoyl *O*-diglucosyl ester	21.972	C30CH36O18	684.2654	683.2654	503.1987; 341.1430; 179.0558	+	−	−
16.	Sesquiterpene	22.919		504.2468	549.2509 [M + HCOO]^-^	503.2468; 463.2046; 410.5768; 371.1986; 325.0901; 251.0740; 149.0441	+	−	−
17.	Quercetin-3-*O*-hexose-deoxyhexose	22.406	C30H26O14	609.1269	609.1196	463.1251; 301.0352; 179.0011; 150.9974; 151.0030	−	+	^32^
18.	Hydroxybenzoic acid isomer 2	23.643	C7H6O3	138.0227	137.0227	93.0335	−	+	^60^
19.	Ferulic acid isomer	24.282	C10H10O4	194.0545	193.0545	161.0258; 134.0380	+	+	^25^
20.	Ferulic acid	25.544	C10H10O4	194.0545	193.0545	161.0258; 134.0370	+	+	^25^
21.	Ferulic acid dihexoside	26.303	C22H30O14	518.1730	517.1730	193.0517	+	−	^28^
22.	3-*O*-glucopyranosyl—glucopyranosyl – arabinopyranosyl—bayogenin 28-*O*-rhamnopyranosyl(1–4)-glucopyranosyl(1–6)-glucopyranosyl ester	27.784	C65H106O33	1414.6677	1413.6677	943.4818; 781.4267; 619.3908; 487.3647; 471.2445	+	−	^18,19^
23.	Chicoric acid isomer 1	28.261	C20H26O13	474.0806	473.0806	311.0349; 179.0360; 149.0092; 135.0442	+	+	^29,62^
24.	3-*O*-glucopyranosyl—glucopyranosyl – arabinopyranosyl – hederagenin 28-*O*-rhamnopyranosyl(1–4)-glucopyranosyl(1–6)-glucopyranosyl ester	28.375	C65H106O32	1398.6615	1397.6515	927.4645; 765.3851; 603.3728; 471.3320	+	+	^18,19^
25.	3-*O*-glucopyranosyl—arabinopyranosyl – bayogenin 28-*O*-rhamnopyranosyl(1–4)-glucopyranosyl(1–6)-glucopyranosyl ester	28.762	C59H96O28	1252.6153	1251.6153	781.4519; 619.3788; 487.3556; 471,0539; 469.1561	+	+	^18,19^
26.	Chicoric acid isomer 2	29.091	C20H26O13	474.0806	473.0806	311.0258; 179.0271; 148.9993; 135.0311	+	+	^29,62^
27.	Pulsatilloside D (3-*O*-glucopyranosyl—arabinopyranosyl – 23-hydroxybetulinic acid 28-*O*-rhamnopyranosyl(1–4)-glucopyranosyl(1–6)-glucopyranosyl ester)	29.407	C59H96O27	1236.6207	1235.6207	765.4164; 603.3685; 471.1480; 469.1372; 451.1337	+	+	^18,19,21^
28.	3-*O*- arabinopyranosyl - bayogenin 28-*O*-rhamnopyranosyl(1–4)-glucopyranosyl(1–6)-glucopyranosyl ester	29.899	C53H86O23	1090.5632	1089.5632	619.3765; 473.4086; 471.1748; 469.1635; 478.4177	+	+	^18,19^
29.	Leonloside D (3-*O*-glucopyranosyl—arabinopyranosyl – hederagenin 28-*O*-rhamnopyranosyl(1–4)-glucopyranosyl(1–6)-glucopyranosyl ester)	30.282	C59H96O27	1236.6207	1235.6207	765.4365; 603.3756; 471.1528; 469.1538; 451.1649	+	+	^14,18,19^
30.	3-*O*-arabinopyranosyl – 23-hydroxybetulinic acid 28-*O*-rhamnopyranosyl(1–4)-glucopyranosyl(1–6)-glucopyranosyl ester	31.265	C53H86O22	1074.5681	1119.5616 [M + HCOO]^-^	1073.5681; 603.3960; 471.11869; 469.1579; 409.1282	+	+	^18,19,22^
31.	Coumaric acid derivative	32.635	C21H28O13	488.0960	487.0960	325.0578; 163.0298	+	+	−
32.	Hederacoside C (3-*O*- arabinopyranosyl- rhamnopyranosyl hederagenin 28-*O*-rhamnopyranosyl(1–4)-glucopyranosyl(1–6)-glucopyranosyl ester) / Anemoside B4 (3-*O*- arabinopyranosyl- rhamnopyranosyl 23-hydroxybetulinic acid 28-*O*-rhamnopyranosyl(1–4)-glucopyranosyl(1–6)-glucopyranosyl ester)	33.025	C59H96O26	1220.6263	1219.6263	749.4374; 471.1673; 469.1582	+	−	^18,21,22^
33.	*O*- arabinopyranosyl – glucopyranosyl 23-hydroxybetulinic acid ester	33.510	C41H66O13	766.4445	811.4601 [M + HCOO]^-^	765.4445; 603.3898; 585.3713; 453.3383	+	−	^18,19^
34.	Dehydrodiferulic acid isomer 1	33.571	C20H18O8	386.0992	385.0992	193.0515; 161.0237; 134.0391	−	+	^58,26^
35.	3-*O*- arabinopyranosyl – hederagenin 28-*O*-rhamnopyranosyl(1–4)-glucopyranosyl(1–6)-glucopyranosyl ester	33.889	C53H86O22	1074.5681	1073.5681	603.3825; 471.1534; 469.1557; 409.1250	+	−	^18,19^
36.	Pulsatilloside C (28-*O*-rhamnopyranosyl(1–4)-glucopyranosyl(1–6)-glucopyranosyl 23-hydroxybetulinic acid ester)	34.334	C48H78O18	942.5085	987.5190 [M + HCOO]^-^	941.5085; 471.3455; 469.1608	+	+	^22,63^
37.	3*-O*- arabinopyranosyl – glucopyranosyl 23-hydroxybetulinic acid ester	35.472	C41H66O13	766.4527	811.4601 [M + HCOO]^-^	765.4527; 603.3868; 585.3713; 453.3383;	+	−	^18,19^
38.	23-hydroxybetulinic acid *O*-rhamnopyranoside	36.694	C36H58O8	618.1762	617.1762	471.1239; 453.1241; 437.0878	+	−	^18,19^
39.	Dehydrodiferulic acid isomer 2	38.050	C20H18O8	386.0992	385.0992	193.0515; 161.0237; 134.0391	−	+	^26^
40.	Hederagenin *O*-rhamnopyranoside	38.484	C36H58O8	618.1762	617.1762	471.1239; 453.1241; 437.0878	+	−	^18,19^
41.	3-*O*- arabinopyranosyl – 28-*O*- glucopyranosyl hederagenin ester	39.864	C41H66O13	766.4403	811.4601 [M + HCOO]^-^	765.4403; 603.3916; 471.3386	+	−	^18,19^
42.	3-*O*- arabinopyranosyl – 28-*O*- glucopyranosyl hederagenin ester	40.361	C41H66O13	766.4434	811.4601 [M + HCOO]^-^	765.4434; 603.3794; 471.3386	+	+	^18,19^
43.	Unknown	49.457		783.5296	782.5296	615.4189; 573.3723; 539.9341; 355.2680	+	+	

**Figure 4:**
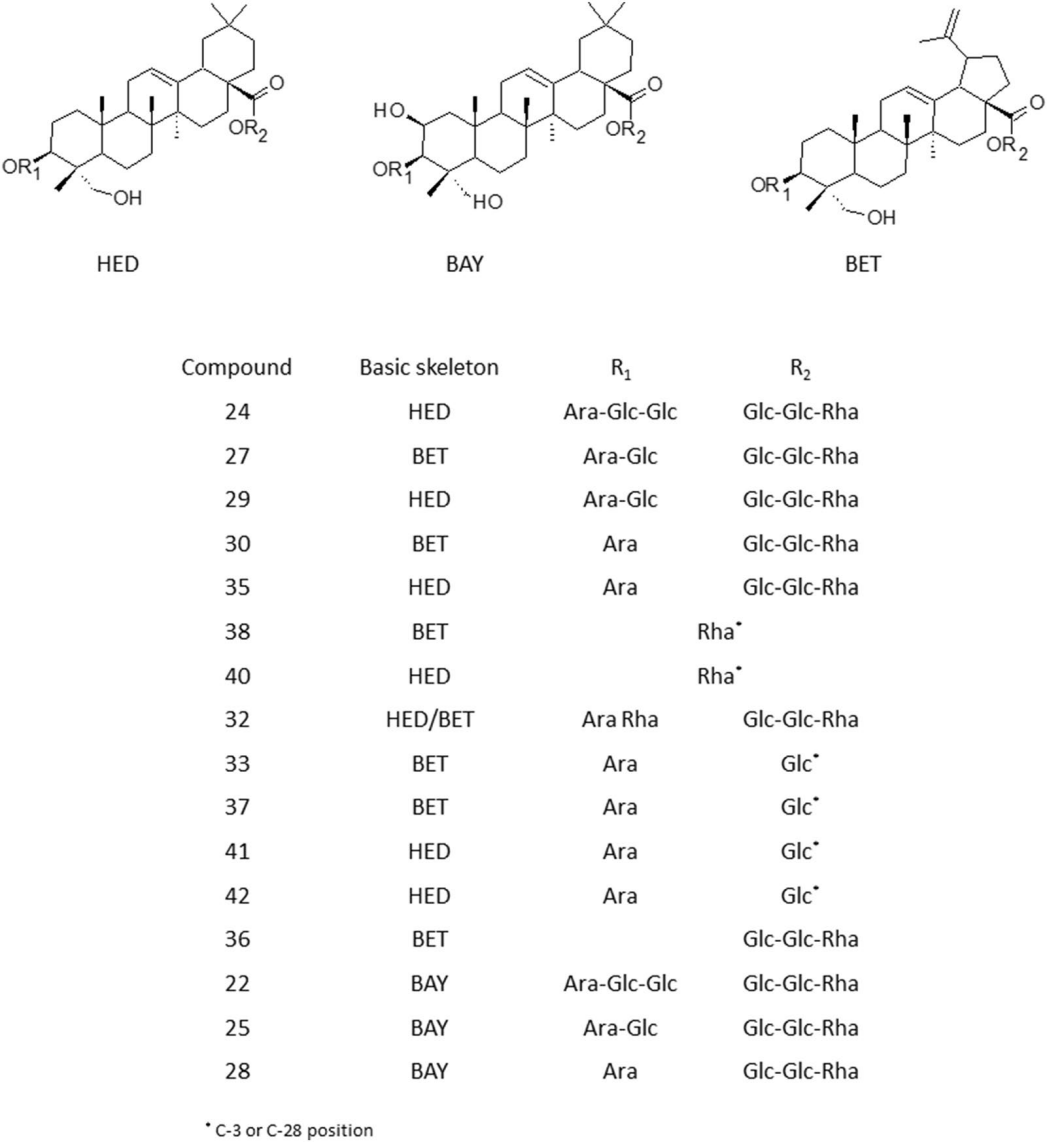
Types of triterpenoid saponins identified in *P. patens* extracts.

*P. patens* contained two sapogenin skeletons, determined as lupane (Lup-type) and oleane-type (Olean-type) (Fig. 4), which produced characteristic fragment ions at *m/z* = 471^19^. These fragment ions may be related to both 23-hydroxybetulinic acid (anemosapogenin) and hederagenin structure what may explain the existence of isomers in the analyzed samples. According to Jin et al*.*^18^ these two types of aglycones can be distinguished by their different retention times resulting from smaller Clog P-value of Lup-type, and consequent earlier elution of Olean-type saponins on the reversed-phase column. Additionally, several peaks shared a similar fragmentation pathway but were assigned to other than hederagenin, olenane-type oligoglycosides, which primarily yielded an abundant ion at *m/z* 487 suggesting the presence of bayogenin structure, reported previously by^20^ in another species *P. patnes* var. *multifida.*

The detailed spectrometric data revealed that peaks **22, 25** and **28** shared the same basic skeleton of bayogenin identified based on the characteristic fragment ion at *m/z* 487, which further generated a product ion at *m/z* 471 as a result of the neutral elimination of H_2_O^19^. The difference between analyzed compounds originated from residues at an α-sugar chain. The observation of precursor ions at *m/z* 943, 781 and 619 for compounds **22**, **25** and **28**, respectively resulted from the elimination of the entire *β*-sugar chain from C-28 position. For compound **22**, based on the product ions with values of 781, 619 and 487, we deduced that two Glc and one Ara molecule were linked to C-3 position. Therefore, the chemical structure of peak number **22** was tentatively established as 3-*O*-glucopyranosyl-glucopyranosyl–arabinopyranosyl-bayogenin 28-*O*-rhamnopyranosyl(1–4)-glucopyranosyl(1–6)-glucopyranosyl ester, while compounds **25** and **28** as 3-*O*-glucopyranosyl- arabinopyranosyl–bayogenin 28-*O*-rhamnopyranosyl(1–4)-glucopyranosyl(1–6)-glucopyranosyl ester and 3-O-arabinopyranosyl-bayogenin 28-O-rhamnopyranosyl(1–4)-glucopyranosyl(1–6)-glucopyranosyl ester, respectively.

Peak **24** produced an abundant precursor ion [M-H]^-^ at *m/z* 1397. Further fragmentation of this molecule generated product ion at *m/z* 927 derived from neutral losses of Glc-Glc-Rha sugar chain from the C-28 position. The MS/MS spectra revealed three products ions at *m/z* valued 765, 603 and 471, due to the sequential loss of Glc, Glc, Ara from C-3 position thus peak **24** was presumed to be 3-*O*-glucopyranosyl–glucopyranosyl–arabinopyranosyl hederagenin 28-*O*-rhamnopyranosyl-(1–4)-glucopyranosyl-(1–6)-glucopyranosyl ester.

Compounds **27** and **29** showed the same formula of C_59_H_96_O_27_ and yielded identical precursor ions [M-H]^-^ at m/z 1235. Compared with peak **24** the molecular weight of these two compounds was 162 Da less, suggesting the lack of one glucose in the *β*-sugar chain. Both **27** and **30** exhibited similar fragmentation patterns. The characteristic fragment ion with *m/z* value of 765 indicated the elimination of the entire Glc-Glc-Rha from C-28. The detection of ions at *m/z* 603 and 471 at compound **27** was attributed to the sequential loss of Glc and Ara, respectively. The identical ions found in both analyzed compounds confirmed the existence of the same sugar chain at C-3. Saponin **27** and **29** were expected to be isomers with different aglycone structure belonging to lupane or hederegenin. The saponin **27** eluted earlier at 29.407 min was characterized as Lup-type and tentatively identified as Pulsatilloside D^18,21^, whereas saponin **29** eluted at 30.282 min as Leonloside D (= leiyemudanoside)^14,18^.

The compound **30** characterized with an adduct ion [M + HCOO]^-^ at *m/z* 1119, gave the precursor ion at *m/z* 1073, which was 162 Da less than that of peak **29**, corresponding to the lack of one Glc group in the *β*-sugar chain of compound **30**. Comparing with the fragmentation of compound **29** and appearance of similar product ions at *m/z* 603 we could deduce that C-28 residues correspond to Glc-Glc-Rha, whereas ions observed at *m/z* 471, resulted from a loss of one Ara moiety from C-3. Thus saponin **30** eluted at 31.265 min was identified as 3-*O*- arabinopyranosyl–23-hydroxybetulinic acid 28-*O*-rhamnopyranosyl(1–4)-glucopyranosyl(1–6)-glucopyranosyl ester. Consequently, its isomer **35** eluted later at 33.889 min was assigned as 3-*O*-arabinopyranosyl–hederagenin 28-*O*-rhamnopyranosyl(1–4)-glucopyranosyl(1–6)-glucopyranosyl ester.

Another pair of isomers **38** and **40,** which produced identical precursor ions at *m/z* 617 have been found. Further fragmentation yielded ion at *m/z* 471 indicating the neutral elimination of Rha moiety from both saponins. In the MS/MS spectra of compounds **38** and **40** the characteristic ion at *m/z* 247, formed from the ring cleavage reaction has been observed. Unfortunately, in this case, data derived from negative-ion mode was insufficient to assume the location of Rha fragment neither in C-3 nor C-28 position. Based on the differences in elution behavior of analyzed compounds, saponin **38** was assigned as Lup whereas **40** as Olean-type and they have proposed to be 23-hydroxybetulinic acid *O*-rhamnopyranoside and hederagenin *O*-rhamnopyranoside, respectively^18^.

Interestingly, four isomeric compounds **33**, **37**, **41** and **42** showed the same molecular formula of C_41_H_66_ O_13_ and afforded an adduct ion [M + HCOO]^-^ at *m/z* 811, which fragmented in slightly different ways. For saponins **41** and **42** the most prominent precursor ion was observed at *m/z* 765 and further fragmentation of [M-H]^-^ formed product ions at *m/z* 603 and 471, which correspond to one Glc (-162 Da) in either C-3 or C-28 and one Ara (− 132 Da) molecule in C-3 position. While for the second pair of isomers **33** and **37** an abundant product ion at *m/z* 603, correlating to neutral loss of Glu, was observed in MS/MS spectrum. The differences in fragmentation behavior of analyzed isomers and their fragment ions abundances might originate not only from aglycone structure but also from the variation in C-3 or C-28 composition. The MS data obtained from negative-ion mode was insufficient to clearly determine the localization of glucose moiety. The elution behavior of analyzed compounds and literature data, indicate the slightly more polar saponins **33** and **37** eluted at 33.510 and 35.472 min, respectively to be Lup rather than Olean-type saponins, and tentatively identified as two isomers of *O*-arabinopyranosyl– glucopyranosyl 23-hydroxybetulinic acid ester, while **41** and **42** isomers were proposed to be hederagenin esters with various glucose location (Fig. 4). According to Jin et al*.*^18^ one of the present isomers **41** or **42** could be tentatively assigned as Plusatilla B (3-*O*-arabinopyranosyl–28-*O*-glucopyranosyl hederagenin ester) previously reported in *P. chinensis* roots.

Compound **36** formed two ion peaks at *m/z* 987 and at *m/z* 941, assigned as adduct ion [M + HCOO]^-^ and precursor ion [M-H]^-^, respectively. Further fragmentation yielded characteristic ion at *m/z* 471, as a result of the typical neutral loss of 470 Da, suggesting that C-28 position was substituted with Glu-Glu-Rha sugar chain. The same fragmentation patterns as in literature data allowed compound **36** to be identified as 23-hydroxybetulinic acid 28-*O*-rhamnopyranosyl(1–4)-glucopyranosyl(1–6)-glucopyranosyl ester, known as Pulsatilloside C^22^.

Compound **32** characterized with precursor ion at *m/z* 1219 and two notable fragment ions at *m/z* 749 and 603, showed a decrease of 146 Da compared to that of saponin **30**, indicating the presence of one Rha moiety (146 Da) in ẞ-sugar chain. The sequence of sugar moieties linked to C-3 position was determined based on literature data^23^. The characteristic product ion at *m/z* 749 might indicate on Anemoside B4 structure also found in *P. chinensis* and *P. koreana*^21–23^. However, in the absence of sufficient data, the compound **32** might be as well tentatively identified as Olean-type saponin, namely Hederacoside C^18^.

Another group of secondary metabolites identified in samples of *P. patens* were phenolic acids. In the category of hydroxycinnamic acid the most abundant were caffeic and ferulic acids derivatives, which occurrence was in accordance with data reported for *P. pratensis* (L.) Mill by^24^.

Compound **20** presented the precursor ion [M-H]^-^ at *m/z* 193 and yielded the fragment ions at *m/z* 178 and 134, attributed to the cleavage of CH_3_ group and characteristic for phenolic acids loss of carbon dioxide from the carboxylic acid group^25^. By comparison with the UV–vis spectra, retention time and fragments generated during CID of the reference standard, compound **20** was unambiguously identified as ferulic acid, while peak **19** was its isomer.

The remaining ferulic acid derivatives were tentatively assigned as ferulic acid glucoside (isomers **13** and **14**) or dihexoside (compound **21**) due to the neutral loss of one or two hexoside moieties from [M-H]^-^ resulting in an abundant fragment ion at *m/z* 193. Similar fragmentation pattern observed for compounds **11**, **13**, **14**, **21, 34, 39** indicated on ferulic acid in their structure. Based on work^26^ compounds **34** and **39** were proposed to be isomers of dehydrodiferulic acids.

The mass spectrum for compound **8** showed the precursor ion [M-H]^-^ at *m/z* 179 and was identified as caffeic acid. Other typical for caffeic acid ions at *m/z* 135 and 161, derived from the decarboxylation and dehydration of caffeic acid as well as ions at *m/z* 107 seen in MS/MS spectrum, was found to be consistent with previously published data^27,28^. The remaining compounds **1**, **5**, **15**, **23** and **26** were tentatively identified as caffeic acid derivatives, according to their MS/MS spectral data. For instance, compounds **1** and **5** generated similar pseudo molecular ion at *m/z* 341. The neutral loss of hexoside moiety (-162 Da) resulted in characteristic for caffeic acid, fragment ion at *m/z* 179. It must be noted that the loss of 162 Da moiety may also result from the neutral loss of single caffeic acid molecule^29^. Thus, compound **1** was tentatively assigned as caffeic acid hexoside while compound **5**, which generates similar fragment ions, as dicaffeic acid^30^. According to fragments observed in MS/MS spectrum of compound **15** such as ions at *m/z* 683, 503, 341 and 179 this molecule was tentatively identified as caffeic acid syryngoyl O-diglucosyl ester.

Another pair of compounds, **23** and **26** producing the same precursor ion [M-H]^-^ at *m/z* 473 with a molecular formula of C_20_H_26_O_13_ was identified. Their MS/MS spectrum revealed the prominent ion at *m/z* 311, due to the neutral loss of a single caffeic acid molecule (-162 Da) and diagnostic fragment ions at *m/z* 179 (caffeic acid), 149 (tartaric acid) as well as *m/z* 135, corresponding to caffeic acid decarboxylation. Because of lack of product ion at *m/z* 341 (relevant for caftaric acid glycoside) in both MS/MS spectra and based on the work^29^, the compounds **23** and **26** could be classified as isomers of chicoric acid (= *di*-caffeoyltartaric acid).

Similarly, compound **7** characterized by precursor ion at *m/z* 311 and a molecular formula of C_13_H_12_O_9_, was tentatively identified as caftaric acid. The tree notable product ions observed in MS/MS spectrum at *m/z* 179, 149 and 135 were in accordance to data reported by^29,30^ for other plant materials.

Among hydroxycinnamic acids, trace amounts of coumaric acid primarily in roots and its derivatives (in leaves) were also found. Compound **6** yielded precursor ion at *m/z* 163 and a molecular formula of C_9_H_8_O_3_. The most prominent product ions were at *m/z* 135 (-28 Da) and 119 (− 44 Da). Regarding presented data, peak **6** was presumed to be coumaric acid. Similarly, for compound **31** the fragmentation of [M-H]^-^ at *m/z* 487 produced two notable product ions at *m/z* 325 and at *m/z* 163. Based on this data compound **31** was tentatively identified as coumaric acid derivative.

The ESI–MS signals for hydroxybenzoic acid derivatives were also identified in analyzed samples, mainly at *m/z* 167, 181 and 137, corresponding to valinic (**10**), hydroxymelilotic (**3**) and hydroxybenzoic acids isomers (**4** and **18**), respectively. Compared with literature data reported by^30^ and spectrum obtained from Human Metabolome Database the fragmentation ions at *m/z* 152 and 108 confirmed the valinic acid structure. In terms of compounds **4** and **18** further fragmentation by CID, revealed the characteristic loss of CO_2_ from its precursor ion, generating the diagnostic ion at *m/z* 93. Due to the identical fragmentation pattern, we deduced compounds **4** and **18** to be isomers of hydroxybenzoic acid. Compound **2** characterized by precursor ion at *m/z* 315 and fragment ions at *m/z* 153, 109 was tentatively identified as dihydroxybenzoic acid hexoside, by comparison with spectral data reported for other plant samples by^27,31^.

Compound **17** generated product ions at *m/z* 463 and 301 from precursor ion [M-H]^-^ at *m/z* 608. It’s characteristic UV–vis spectra (λ_max_ 257, 357 nm) as well as detected in MS/MS spectrum diagnostic fragment ions at *m/z* 179 and 151 supported the existence of a quercetin aglycone. So the chemical structure of compound **17** was tentatively identified as quercetin-3-*O*-hexose-deoxyhexose^32^.

According to^33^ the anemonin structure was also reported in the roots of *P. patens*. The peak **9** was characterized with a molecular formula of C_10_H_8_O_4_ and fragment ions at *m/z* 191, 147 and 101.

Unfortunately, some of the constituents could not be identified **12**, **43** and **18** (sesquiterpene) because of the insufficient data.

## Discussion

Uncontrolled cell division, escaping the external and internal control of the cell cycle, leads to unlimited proliferation (multiplication) of cancer cells and neoplastic processes (neoplasia, carcinogenesis)^34^. This is often due to mutations in DNA regions encoding genes important for cell cycle control mechanisms. A panel of 13 vectors (transcription factors) induced by the luciferase reporter gene was used in our research. These vectors enable efficient expression of the protein encoded by the luciferase reporter gene in the system under investigation. Their expression was carried out by enhancer elements (inducers) (such as, IL6, TGF-β, PMA, Wnt 3a) that bind to specific transcription factors. The vectors and inducers included in this study and the studies of other authors^17,35^ are used to assess the activity of most of the cancer-related signaling pathways. Similarly [(E)-3,5,4′-trihydroxystilbene], a parent compound of the analog used in this study, which is present in a wide range of plants, such as *Arachis hypogaea* L. (Fabaceae) or *Vitis vinifera* L. (Vitaceae), is used as a positive control^35^. It is the type of natural phenol, and a phytoalexin produced by several plants in response to injury or when the plant is under attack by pathogenic bacteria and fungi. Resveratrol is the most well-known stilbene, which is postulated as a potential modulator of signal transduction pathways for cancer and the carcinogen response^35^.

The modulation of cancer-related signaling pathways and determining the mechanism of action of transcription factors on the inhibition of the neoplastic process is an important topic in recent years.

STAT3 (Signal Transducers and Activators of Transcription) is a protein, involved in the transmission and the activation of gene transduces from the STAT protein family. In the Stat3 signaling pathways, cytokine IL-6 used as an inducer in our research leads to its activation (Janus kinase JAK) (Fig. 3). Stat3 is phosphorylated by the receptor^36^ and translocated to the nucleus, where it promotes transcription of target genes, including prooncogenetic ones^37^. The inhibition of STAT3 in HeLa cell signaling caused by methanolic extract of *P. patens* is associated with the inhibition of growth and inhibition of cell proliferation and their apoptosis (Fig. 3).

Smad is a signaling pathway activated by TGF-β and taking part in the regulation of target gene expression (Fig. 3). In our study, in Smad/ TGF-β signaling pathway, the ligand TGF-β was bound and connected with two pairs of type I and type II receptors, which are transmembrane Ser/Thr kinases. In these receptor complexes, the type II receptor phosphorylated and activated the type I receptor kinase, which then phosphorylated and activated Smad. The Smad/TGF-β signal transduction pathway is important in signaling and regulation of cells functions during the whole life of the organism^38^.

The MYC—is an induced nuclear protein antigen, encoded by the MINA gene. MYC is a transcription factor involved in proliferation, growth, and cellular development, and direct regulation of target genes. MYC is controlling the complex networks of microRNAs and apoptosis mediators^39^. In this study, MYC was activated by PMA (protein kinase activator), which is phorbol 12-myristate-13-acetate (Fig. 3). As reported previously by several researchers, MYC can be activated by the direct interaction with the acetyltransferases p300 and CBP^39^. In our modulation of cancer-related signaling pathways, many transcription factors were activated by PMA (protein kinase activator). These are NF-κB (nuclear factor kappa-light-chain-enhancer of activated B cells), AP-1 (Activator Protein 1), Ets, Hedgehog, and Notch (Fig. 3). NF-κB can also be activated by the enzyme IκB kinase and then translocated into the nucleus. The Notch can be activated by specific Serine/threonine (Ser/Thr) protein kinase and E2F by—Cyclins, cyclin-dependent kinases CDKs connected with the cyclin-dependent kinase inhibitors (p15, p16). This article reports the use of the methanolic extract of the *P. patens* which inhibits the progression of cancer through the modulation of cancer-related metabolic signaling pathways. Numerous studies support a pro-oncogenic function for Notch signaling too. It was reported that the modulation of the Notch pathway is important in controlling the development of cancer cells and has bidirectional complicated interaction with multiple other pathways that include candidate therapeutic targets^40^. Same for the Ets family (Erythroblast Transformation Specific) which are one of the largest families of transcription factors to be associated with cancer, such as through gene fusion^41^. Their deregulation following changes in expression and transcriptional activity plays a critical role in carcinogenesis (neoplasm, tumor formation), apoptosis (programmed cell death), and neoplastic angiogenesis by the ability to form blood vessels within a neoplastic tumor enables further growth tumor mass. All ETS family members are identified through a highly conserved DNA binding domain, the ETS domain, which is a winged helix-turn-helix structure that binds to DNA sites with a central GGA (A/T) DNA sequence^38^.

The E2F family comprises of basic helix-loop-helix transcription factors that supervise the expression of genes associated with the regulation of the cell cycle^42^. The transcriptional targets of E2F include cyclins, cyclin-dependent kinases, checkpoints regulators, DNA repair, and replication proteins (Fig. 3). The cancer-related proliferative roles of E2F family members represent a recent evolutionary adaptation^42^. The evolutionarily conserved Hedgehog (Hh) signaling pathway (synonyms: Hedgehog-Patched (Hh-Ptch), Hedgehog-Patched-Smoothened (Hh-Ptch-Smo)) is responsible for signal conveyance from the cell membrane into the nucleus (Fig. 3). The Hh signaling pathway plays a significant role in normal embryonic development and the maintenance of stem cells important for tissue repairs, such as mammary, skin, neural, lung stem cells, and some epithelial cells of internal organs^43^.

Activator protein 1 (AP-1) is a transcription factor responsible for the regulation of gene expression in reaction to various stimulants, including viral and bacterial infections, growth factors, cytokines and stress. AP-1 controls proliferation, differentiation and apoptosis^44^. A variety of extracellular matrix and genotoxic agents were shown to induce AP-1 activity, which supports the involvement of AP-1 in programmed cell death.

The FoxO transcription factors oversee the protection from oxidative stress by regulating antioxidants and controlling the protein quality. If the expression or activation of FoxO becomes dysregulated it may lead to a variety of age-related disorders affecting CNS (central nervous system) bones, muscles, or leading to cancer^45^. Moreover, the FoxO regulates the cell cycle, the proliferation of cells and apoptosis.

Protein Wnt-3a is encoded by the WNT3A gene^46^ which belongs to the WNT gene family. The WNT includes a series of genes that encode secreted signaling proteins (Fig. 3) which are involved in adipogenesis, oncogenesis, and the regulation of embryogenesis. WNT3 and WNT3A genes are two human paralogues of mouse proto-oncogene Wnt3, which induces carcinogenesis through activation of the β-catenin—TCF signaling pathway^46^.

Reporter genes were introduced by transfection into cells grown in cell culture (Table 5). The inhibition of the progression of human cancer cells by many cancer-related signaling pathways were studied on the species *Pulsatilla patens* (L.) Mill. The *Pulsatilla* species is a good candidate as a natural product for use in the treatment of cancer. This is indicated by the research of other authors about *P. koreana* Nakai^16^ or *P. chinensis* (Bunge) Regel^47^*.* In the study with *P. koreana* it was found that SB365, Pulsatilla saponin D isolated from the root of this species, strongly suppressed the growth and proliferation of colon cancer cells and induced their apoptosis. Additionally, SB365 showed antiangiogenic activity and significantly inhibited tumor growth^16^. In anticancer mechanism, SB365 effectively suppressed the AKT/mTOR pathway both in vitro and in vivo. Moreover, deoxypodophyllotoxin (DPT) from the extract of *P. koreana* was found as a cytotoxic and antiangiogenic component combined with antitumor activity. The DPT from the extract of *Pulsatillae* Radix which exhibited antiangiogenic activity and good antitumor activity against mice bearing Lewis lung carcinoma (LLC) is an active constituent of SB31^48^. The biologically active compounds isolated from the root extract of *P. koreana* have been found to show antibacterial, antiparasitic and anti-inflammatory^49^ activities too. Other species *P. patens* and *P. vulgaris* from our previous research showed antibacterial and antifungal activities^50^. *P. chinensis* has been found to show antitumor activities too. Pulsatilla saponin A from this species significantly inhibited the growth of human hepatocellular carcinoma SMCC-7721 cells and pancreatic BXPC3 and SW1990 cancer cells^47^. Similar inhibitory activities were observed when the compound was tested in mouse xenograft tumor models using human hepatocellular carcinoma Bel-7402 and pancreatic cancer SW1990 cells. Pulsatilla saponin A may exert its antitumor effect by inducing DNA damage and causing G2 arrest and apoptosis in cancer cells^47^. The triterpene glycosides (Pulsatilla saponins A and B) and the triterpene acid isolated from *P. chinensis* shows high cytotoxic activity against the malignant cells of the lung cancer^21^ and are used for antibacterial, antiparasitic, antiprotozoal, antifungal and molluscicidic^51^ activities. Our study about the active compounds of methanolic extracts of *P. patens* is very important too because these are the first studies of *Pulsatilla patens* species which its antitumor properties in the luciferase reporter test performed.

**Table 5:** The panel of 13 inducible luciferase reporter gene vectors where expression is driven by enhancer elements (Inducer) that bind to specific transcription factors.

Name of the vector	Inducer (Alternate inducer)	Fold of induction	Duration of treatment
STAT3	IL6	15x	4-h induction
SMAD3/4	TGF-β	15x	
NF-κB	PMA	20x	
AP-1	PMA	50x	
Ets	PMA	8x	
E2F	PMA	4x	
MYC	PMA	7x	
pTK control	No inducer	Not applicable	
Notch	PMA	4x	6-h induction
FoxO	No inducer	Not applicable	
Wnt	Wnt-3a	4x	
Hedgehog	PMA	20x	
miR-21	No inducer	Not applicable	
k-Ras	No inducer	Not applicable	

There are various classes of plant secondary metabolites influencing cancer-related signaling pathways. Triterpenoid saponins, naphthoquinones, chalcones, sesquiterpene lactones, curcuminoids, flavonoids and isothiocyanates are selective for cancer cells because they are much more active within cancer cells than in normal cells (Supplementary Table S2). Transformed cells are less able to handle the oxidative stress induced by these compounds and therefore redox-sensitive targets are more greatly impacted. These classes of compounds are great for targeting cancer-relevant signaling pathways in cancer cells and play a major role in reactive oxygen species (ROS) generation^52^, the depletion of the antioxidant glutathione (GSH), target redox reactions, and redox-sensitive cysteine^53^.

The Authors hereby would like to point out the limitation related to the presented results. The suggested influence of *P. patens* on apoptosis was based on the studies of cancer-related signaling pathways in HeLa cells, being the main goal of our research, and not on the direct measurements of apoptosis. We acknowledge that the potential proapoptotic activity of *P. patens* requires additional research focusing on apoptosis with the use of direct measurement of cellular caspases (caspase-3, -7, -8 or -9), DNA fragmentation, and Annexin V based assays using fluorescence microscopy or flow cytometry.

Our phytochemical analysis of the methanolic extracts of the *P. patens* shows that compounds, which strongly suppressed the growth and proliferation of HeLa cancer cells are mainly saponins. The triterpenoid saponins and phenolic acids were characterized and identified from a methanolic extract of the *P. patens* roots and leaves. Similar biological properties of saponins of the genus *Pulsatilla* in the treatment of cancer and other diseases together with *P. koreana*^54^ and *P. chinensis*^47^ has been shown for other species such as *P. cernua* (Thunb.) Bercht. et Opiz. Too^55^, *P. nigricans* Storck^56^, *P. dahurica* (Fisch. ex DC.) Spreng.^57^, *P. turczaninovii* Kryl. et Serg.^58^ or *P. pratensis* (L.) Mill.^24^. Saponins from *Pulsatilla* spp. have demonstrated multiple biological properties including antitumor^59^, neuroactive, immunomodulatory, antioxidant, cognition-enhancing and neuroprotective^55^ activities. In our research was found that active compounds resulted in inhibition of cell growth/proliferation and induction of apoptosis of HeLa cells—cervical cancer. The saponins isolated from the methanolic extract of *P. patens* subsp. *multifida* (G.A. Pritel) Zämelis, the native species from the USA and Asia, have been used for ages in traditional medicine as the agent showing anticancer properties, inhibiting the growth of skin cancer^20^. But no one until now has identified the antitumor properties of *P. patens* subsp. *patens* of a native species from Europe using cancer-related signaling pathways in HeLa cells.

## Conclusion

Research on the methanolic extracts of *P. patens* confirms strong inhibition of signaling pathways in HeLa cells, a cervical cancer cell line, which may also be of importance for the chemotherapeutic ability in other types of cancer. A characteristic feature of active compounds from *P. patens* is their activity at many levels of cell signaling. In this study it was found, that the root extract of *P. patens* is the most potent in inhibiting the activation of Stat3, Smad, AP-1, NF-κB, MYC, Ets, Wnt, and Hdghog. The pleiotropic nature of this extract is in line with current trends in oncological pharmacology, where it is to replace drugs, in favor of multidirectional therapies. The methanolic extracts of *P. patens* enhanced apoptotic death of HeLa cells, deregulated cellular proliferation, differentiation, and progression towards the neoplastic phenotype by altering key signaling molecules required for cell cycle progression. Since the proapoptotic activity of *P. patens* was observed during studies of cancer-related signaling pathways in HeLa cells the future studies should be focused on the detailed analysis of apoptosis using alternative methods, ex. direct measurement of cellular caspases. This is the first study to report the influence of *Pulsatilla* species on cancer signaling pathways. Effects of the methanolic extracts of *P. patens* in these signaling pathways identified here should be further evaluated in tumor-bearing mice and in the next step in preclinical studies of cervical cancer patients, treated with active compounds from this species. The results showed that *P. patens* is a rich source of polyphenolic constituents, mainly saponins, and hydroxycinnamic acids, wherein their triterpenoid saponins showed a different profile in comparison to other species from the Ranunculaceae family. Our research will be helpful to determine the relevance of each cancer-related signaling pathways that may be used to develop novel therapies that combine extracts of *P. patens* with other agents including STAT, SMAD, AP-1, NF-κB, MYC, Ets, Wnt, and Hdghog blockers, to effectively treat cervical cancer, and other cancers that utilize these pathways.

## Methods

### Plant material

The leaves and roots of *P. patens* were collected in May 2016–2018 at the Knyszynska Forest, Podlaskie Province, in North-Eastern Poland. Plant material was identified by Prof. Grażyna Łaska from the Bialystok University of Technology. In Poland, species *P. patens* has been strictly protected since 1958 and still requires active protection. Collection of plant material for testing was done in accordance with Decision No WPN.6400.23.2016.MW of 27.04.2016 issued by the Regional Directorate of Environmental Protection in Bialystok, 23 Dojlidy Fabryczne Street, 15–554 Bialystok. In order to obtain a larger amount of plant material for future studies, a cooperation agreement was signed between the Bialystok University of Technology and Botanical Garden "Herbal Corner". Within this collaboration, whole plant species were transferred from the natural environment to the Botanical Garden for their propagation. . The use of plants parts in the present study complies with international, national guidelines. The plant material in the form of dry roots (23.6 g) and leaves (32.3 g) was extracted by accelerated solvent extraction (ASE) technique (SpeedExtractor E-916, Buchi) with 80% methanol. The extraction time was 30 min., the temperature was 100 °C and the pressure was 120 bar. Three extracts were prepared from each raw material. Extracts were filtered through paper filters. Methanol was dried using a rotor evaporator. The obtained extracts were subjected to detailed phytochemical analysis.

### Preparation of reference compounds solutions

To provide the quantitative analysis of saponins and hydroxycynnamic acid type compounds two reference substances, pulsatilla saponin D and ferulic acid were used. The pulsatilla saponin D was obtained from TRC-Canada (Toronto, Canada) with 90% purity, while ferulic acid was purchased from Sigma-Aldrich (Madrid, Spain). All standards were precisely weighed and individually dissolved in methanol HPLC‐grade (J.T. Baker, Deventer, The Netherlands) to achieve stock solutions of 1 mg/mL and stored at 4^0^C.

### Conditions for Qualitative and Quantitative LC-ESI-QTOF-MS analysis

The LC-ESI-QTOF-MS analysis was done on Agilent 1200 Infinity HPLC coupled to Agilent 6530BQTOF Accurate-Mass QTOF system equipped with Dual Agilent Jet Stream spray source (ESI) (Agilent Technologies, Santa Clara, CA, USA) and operated with N_2_ generator (Parker Hannifin Corporation, Haverhill, MA; generating a nitrogen supply at purities > 99%). For the chromatographic separation Gemini column (3 μm i.d. C18 with TMS end-capping, 110 Å, 100 × 2 mm) supported with a guard column (PhenomenexInc, Torrance, CA, USA) was used. Chromatographic and spectrometric conditions followed our previous report^31^. The MS spectra acquired in the negative mode provided greater molecular ion abundances and better sensitivity compared to the positive-ion mode. Compounds were tentatively identified based on fragmentation patterns and supported by comparison of obtained mass spectra with mass spectra obtained for reference compounds, mass spectra found in the literature and records available in Metlin database (https://metlin.scripps.edu). Quantification was done based on the UV spectra recorded in 210 and 320 nm for saponins and hydroxycynnamic acid type compounds, respectively.

### Method validation

#### Calibration curves, the limit of detection and quantification

The method linearity was checked by evaluation of the dilutions prepared from stock solutions of reference substances. The construction of calibration curves was based on seven for pulsatilla saponin D, and on eight for ferulic acid concentrations, prepared in triplicate. The detected peak areas were correlated with the corresponding concentrations of reference compounds. The limits of detection (LOD) and limits of quantification (LOQ) under the chromatographic conditions were separately calculated as a signal-to-noise ratio (S/N) of 3 and 10, respectively according to The International Council for Harmonisation of Technical Requirements for Pharmaceuticals for Human Use (ICH) guidelines.

#### Precision and repeatability

The relative standard deviation (RSD) was taken as a measure of precision and repeatability. The intra- and interday variance were measured to obtain the precision of the LC-QTOF-MS method using the standard solutions at two different concentration levels. Each concentration was injected in three replicates on the same day and further on three consecutive days for the determination of the intraday and interday precision, respectively. To investigate the repeatability, five solutions at one concentration were extracted and analyzed.

#### Recovery

To evaluate the accuracy of the method applied, a recovery test was done by spiking the reference solutions to the *P. patens* sample and measuring the concentrations in triplicate.

#### Cytotoxicity assays

The assessment of cytotoxicity was made using cell lines obtained from the ATCC (American Type Culture Collection Rockville, MD, USA) in accordance with the methodological procedure used in the National Center for Natural Products Research (University of Mississippi, MS, USA). The cell line panel included five human cancer cell lines—epidermal carcinoma (KB), breast carcinoma (BT-549), ovarian adenocarcinoma (SK-OV-3), skin malignant melanoma (SK-MEL), cervical adenocarcinoma (HeLa) and two noncancerous kidney cell lines – epithelial cells (LLC-PK-1), fibroblast (Vero). Briefly, cells were passaged (25,000 cells/well) into 96-well microplates (tissue culture-treated) and incubated for 24 h. Afterward, different concentrations of the extract were added and incubation continued for 48 h. Finally, after incubation, a modified Neutral Red assay was used to determine the cell viability^64^. The IC_50_ values were calculated from dose–response curves. The doxorubicin was used as a reference cytotoxic drug (positive control), whereas DMSO was used as a negative control (vehicle control).

#### Transfection and luciferase assays

HeLa cells (ATCC, Bethesda, MD, USA) were maintained in Dulbecco’s modified Eagle’s medium (DMEM) (Gibco Life Technologies, Grand Island, NY, USA) containing 10% fetal bovine serum (FBS, Atlanta Biologicals Inc., Atlanta, GA, USA) and then were plated in white opaque 384 well plates at a density of 4300 cells/well in 30 µL of growth medium supplemented with 10% FBS and 1% Pen/step. The next day, the medium was aspirated and replaced with DMEM containing 10% FBS only. The cells were transfected with respective plasmids using X-tremeGENE HP transfection reagent (Roche Applied Science, Indianapolis, IN, USA). Luciferase vectors used in this assay are summarized in Table 5. Luciferase reporter constructs were transfected into Hela cells as previously described^35^. After 24 h of transfection, the test agents were added to the transfected cells, followed 30 min later by an inducing agent: IL-6 (50 ng/mL, R&D Systems, Inc., Minneapolis, MN, USA) for Stat3^35^, TGF-beta (5 ng/mL, R&D Systems, Inc., Minneapolis, MN, USA) for Smad^35^, Wnt-3a (500 ng/mL, Peprotech Corporation, Rocky Hill, NJ, USA) for Wnt^35^and PMA (phorbol 12-myristate 13-acetate, 77 ng/mL, Sigma Chemical Company, St. Louis, MO, USA) for AP-1, NF-κB, E2F, MYC, Ets, Notch and Hdghog. No inducer was added for FoxO, miR-21, k-Ras and pTK-control (thymidine kinase promoter). After 4 h or 6 h of induction, the cells were lysed by the addition of the One-Glo luciferase assay system (Promega Corporation, Madison, WI, USA). The light output was detected in a Glomax Multi + detection system with Instinct Software (Promega Corporation, Madison, WI, USA). This luciferase assay determines if the test agent was able to inhibit the activation of cancer-related signaling pathways. In the case of FoxO and mi-R21 enhanced luciferase activity by the test agent was assessed^17,35^.

## Supplementary Information

Supplementary Information.
