# Supplementary material for: Extracts from Pulsatilla patens target cancer-related signaling pathways in HeLa cells
Source: Sci Rep. 2021 May 20;11:10654. doi: 10.1038/s41598-021-90136-3 (PMC8138020; doi:10.1038/s41598-021-90136-3)
Supplement: Supplementary file 1 — Supplementary Information. [file 41598_2021_90136_MOESM1_ESM.doc]

**Supplementary material**

**Extracts from *Pulsatilla patens* target cancer-related signaling pathways in HeLa cells**

**Grażyna Łaska**^1^**, Magdalena Maciejewska-Turska**^2^**, Elwira Sieniawska**^2^***, Łukasz Świątek**^3^**, David S. Pasco**^4^ **and Premalatha Balachandran**^4^

^1^Department of Agri-Food Engineering and Environmental Management, Bialystok University of Technology, Bialystok, 15-351, Poland

^2^Department of Pharmacognosy, Medical University of Lublin, Lublin, 20-093, Poland

^3^Department of Virology, Medical University of Lublin, 20-093 Lublin, Poland

^4^National Center for Natural Products Research, School of Pharmacy University of Mississippi, University, MS 38677, USA

* Corresponding author, e-mail address: [esieniawska@pharmacognosy.org](mailto:esieniawska@pharmacognosy.org)


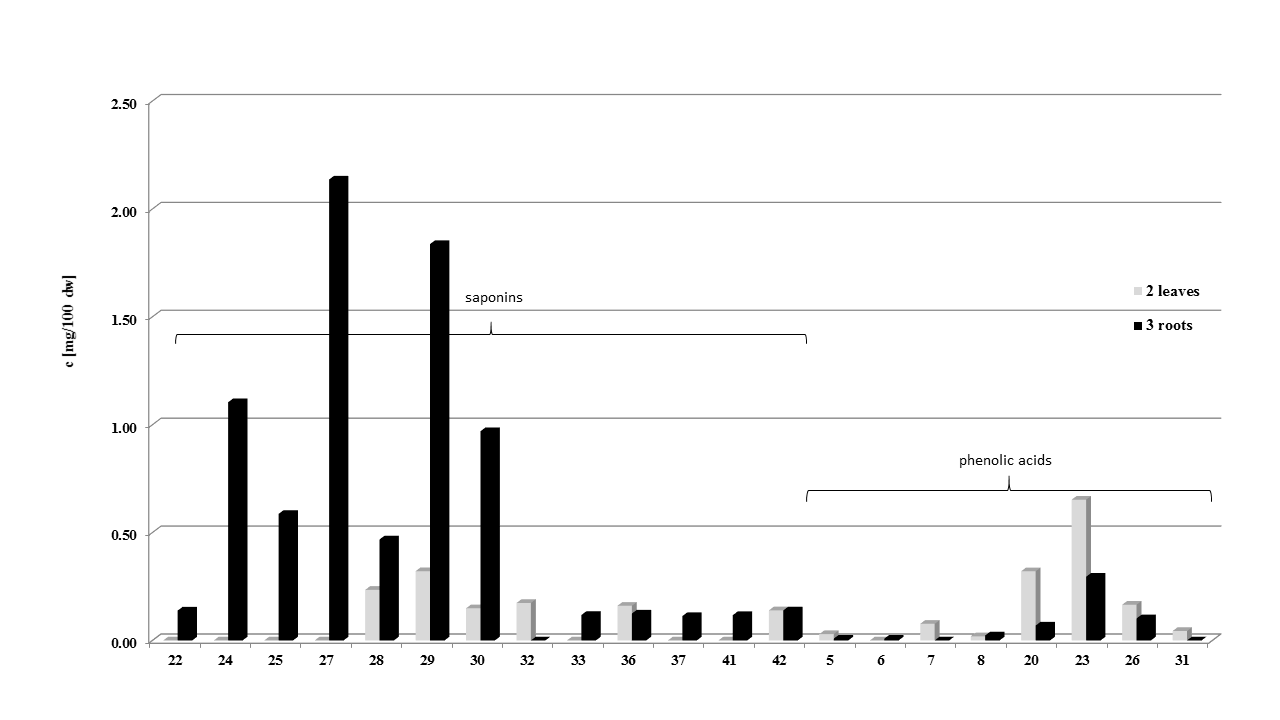


**Supplementary Figure S1.** Content of saponins and hydroxycynnamic acid type compounds in *P. patens* extracts

**Supplementary Table S1.** Results of tests of biological activity of secondary metabolites isolated from methanolic extract of roots (mR) and leaves (mL) of *Pulsatilla patens* (L.) Mill. using their different volumes: 40 µg/mL (test I) and 30 µg/mL and 15 µg/mL (test II), in the presence of various inducers on human cervical carcinoma HeLa cell lines with 13 different vectors (transcription factors)

|  | **Transcription factors (vectors) * in the presence of various inducers ** in the luciferase reporter gene assay** | | | | | | | | | | | | | |
| --- | --- | --- | --- | --- | --- | --- | --- | --- | --- | --- | --- | --- | --- | --- |
| Sample data | Stat3/ IL-6 | Smad/ TGF-β | AP-1/ PMA | NF-κB/ PMA | E2F/  PMA | MYC/  PMA | Ets/ PMA | Notch (CSL-Luc) | FoxO  in 10% FBS | Wnt/  wnt-3a | Hdghog/  PMA | pTK (4h) | miR-21 | k-Ras |
| **Resveratrol analog (6 µM)** | 92 | 68 | 78 | 28 | 64 | 47 | 62 | 65 | 66 | 44 | 57 | 95 | 78 | 86 |
| ***P. patens***  **(mL 40 µg/mL) - I assay** | 103 | 169 | 93 | 45 | 135 | 105 | 126 | 133 | 122 | 126 | 134 | 155 | 169 | 140 |
| ***P. patens***  **(mR 40 µg/mL) - I assay** | 15*** | 14 | 20 | 10 | 31 | -6 | 14 | 57 | 149 | 2 | 24 | 89 | 220 | 62 |
| ***P. patens***  **(mR 30 µg/mL) - II assay** | 37 | 27 | 42 | 19 | 52 | 12 | 34 | 63 | 131 | -6 | 43 | 104 | 203 | 68 |
| ***P. patens***  **(mR 15 µg/mL) - II assay** | 77 | 73 | 63 | 53 | 81 | 46 | 78 | 129 | 124 | 40 | 86 | 133 | 213 | 118 |

**Explanations to the table:**

*** Transcription factors, vectors in the signal transduction pathways of cancer processes** - Stat3, Smad, AP-1, NF-κB, E2F, MYC, Ets, Notch, FoxO, Wnt, Hdghog, miR-21, k-Ras and pTK - control

****** **Inducer, promotor** of cancer processes - IL-6, TGF-β, PMA, wnt-3a

*** - Numerical value expressing the percentage ratio of the activity of the tested samples (metabolites) to the activity of the promoter based on the determination of the activity of the protein encoded by the luciferase reporter gene.

The lower the numerical value in the table, the greater the activity of the test sample is in inhibition than that of the tumor promoter.

Resveratrol analog – compounds with antitumor activity used in the research (explanations in the text)

*P .patens* (mL 40 µg/mL) – is the volume of methanolic extract from leaves of the *Pulsatilla patens* used for the tests - a test I - not biologically active

*P. patens* (mR 40 µg/mL) – is the volume of methanolic extract from the root of the *P. patens* used for the tests - test I - biologically active antitumor

*P. patens* (mR 30 µg/mL) – is the volume of methanolic extract from the root of the *P. patens* used for the tests - test II - biologically active antitumor

*P. patens* (mR 15 µg/mL) – is the volume of methanolic extract from the root of the *P. patens* used for the tests - test II - biologically active antitumor

**Supplementary Table S2.**  Different classes of compounds related signaling pathways (Source: Balachandran et. al., 2014 , changed )^1^

| Compounds | Stat3/ IL-6 | Smad /TGF-β | Ap-1 /PMA | NF-kB/  PMA | E2F/ PMA | Myc/ PMA | Ets/ PMA | Notch/ PMA | FoxO | Wnt  wnt-3a | HdghogP /PMA | pTK (4h) | miR-21 |
| --- | --- | --- | --- | --- | --- | --- | --- | --- | --- | --- | --- | --- | --- |
| **Naphthoquinones** |  | | | | | | | | | | | | |
| Plumbagin | 4.3 | 11.0 | - | 2.5 | - | 12.0 | 12.0 | - | - | - | - | - | - |
| Alkanin | 2.4 | 3.1 | 9.2 | 1.0 | 7.0 | 3.2 | 6.4 | 4.0 | - | 7.0 | 6.0 | - | - |
| Thymoquinone | 15 | 17 | - | 13 | 20 | 18 | - | - | - | 25 | 25 | - | - |
| **Chalcones** |  | | | | | | | | | | | | |
| Butein | 4.1 | 3.8 | - | 3.2 | - | 7.0 | 11.6 | 11.7 | - | 10.0 | 10.6 | - | - |
| Flavokawain B | 8.0 | 15.0 | 21.0 | 3.0 | 12.4 | 15.0 | 16.0 | 23.0 | - | 11.2 | - | - | - |
| Isorliquirtegenin | 8.0 | 12.8 | 25.0 | 8.5 | - | 17.7 | 25.0 | - | - | 21.3 | 26.0 | - | - |
| Flavokawain A | 20.0 | 25.0 | - | 16.7 | - | - | 26.0 | - | - | 18.8 | 26.0 | - | - |
| **Curcuminoids** |  | | | | | | | | | | | | |
| Curcumin | 15 | 9 | - | 7 | - | 12 | - | - | - | 13 | - | - | - |
| CDF | 3.0 | 1.9 | 2.3 | 1.4 | - | 2.7 | 2.7 | 3.1 | - | 2.3 | - | - | - |
| **Sesquiterpene lactones** |  | | | | | | | | | | | | |
| Parthenolide | 3.4 | 7.2 | - | 1.0 | - | 9.0 | 9.9 | - | - | 4.4 | 10.0 | - | - |
| Repin | 2.5 | 6.0 | 6.7 | 0.7 | 8.1 | 5.8 | 6.8 | 6.8 | - | 6.4 | 6.1 | - | - |
| **Isothiocyanates** |  | | | | | | | | | | | | |
| Benzyl isothiocyanate | - | 12 | - | 7 | - | 12 | - | 12 | - | 15 | - | - | - |
| DL-Sulforaphane | 12 | 23 | - | 10 | - | 19 | - | - | - | - | - | - | - |
| Phenethyl isothiocyanate | - | - | - | 8 | 12 | 12 | 18 | 20 | - | - | - | - | - |
| 4-phenyl butyl isothiocyanate | - | - | 20 | 13 | 14 | 19 | 13 | 17 | - | 17 | - | - | - |
| **Flavonoids** |  | | | | | | | | | | | | |
| Fisetin | 19.0 | 12.0 | 9.7 | 15.8 | 18.5 | 10.0 | 12.4 | - | - | 11.7 | 16.2 | - | - |
| Apigenin | 25 | 17 | - | 21 | - | 21 | 23 | - | - | 21 | - | - | - |
| Quercetin | 24 | 10 | 26 | 29 | - | 23 | 26 | - | - | 22 | - | - | - |
| **Saponins** |  | | | | | | | | | | | | |
| Hederagenin | 15 | 14 | 20 | 10 | 31 | -6 | 14 | 57 | 149 | 2 | 24 | 89 | 220 |

**Naphthoquinones**

**Chalcones ROS generation**

**Curcuminoids Target redox Flavonols Target ATP binding**

**Sesquiterpene lactones Deplete GSH Flavones Site of proteins**

**Isothiocyanates Sensitive cysteine**

**Saponins Residues in proteins**

**References**

1. Balachandran, P., Zhang, J., Ibrahim, M. A., Ilias, M., & Pasco, D. S. UMMC-Cancer Institute /NCNPR. Cancer Drug Discovery Core. Annual Meeting of the American Society of Pharmacognosy, 14th Oxford International Conference on the Science of Botanicals, Oxford, Mississippi (2014)
